# Treatment patterns and cost estimations of systemic chemotherapy for pancreatic cancer in Japan: A retrospective database study

**DOI:** 10.1002/cam4.6100

**Published:** 2023-05-18

**Authors:** Yuki Takumoto, Ryotaro Shibahara, Hajime Asami, Manabu Akazawa

**Affiliations:** ^1^ Department of Public Health and Epidemiology Meiji Pharmaceutical University Tokyo Japan; ^2^ Center for Outcomes Research and Economic Evaluation for Health National Institute of Public Health Saitama Japan

**Keywords:** cost estimation, Japan, pancreatic cancer, utilization

## Abstract

**Background:**

This study aimed to clarify the treatment patterns of pancreatic cancer patients receiving systemic chemotherapy in Japan and to estimate the direct medical costs in actual practice.

**Research Design and Methods:**

This retrospective cohort study used electronic health record data between April 2008 and December 2018 in Japan. Participants had a confirmed pancreatic cancer diagnosis and received at least one systemic chemotherapy, including FOLFIRINOX, gemcitabine plus nab‐paclitaxel, gemcitabine, and S‐1. The outcomes were treatment patterns and monthly medical costs and the distribution of monthly medical costs across healthcare resource categories.

**Results:**

Of the 4514 selected patients, 40.7%, 7.1%, 24.4%, and 21.3% used gemcitabine plus nab‐paclitaxel, FOLFIRINOX, gemcitabine, and S‐1 as first‐line chemotherapy, respectively. The median monthly medical costs were the highest in the first month, with gemcitabine plus nab‐paclitaxel ranking first (6813 USD), followed by FOLFIRINOX, gemcitabine, and S‐1. The health resource categories with the highest shares of monthly medical costs during the first‐line treatment period with gemcitabine plus nab‐paclitaxel and FOLFIRINOX were hospitalization costs (FOLFIRINOX: 41%–37%; gemcitabine plus nab‐paclitaxel: 40%–34%) and medicine costs (FOLFIRINOX: 51%–42%; gemcitabine plus nab‐paclitaxel: 49%–38%).

**Conclusions:**

This study sheds light on the current treatment patterns and direct medical costs of systemic chemotherapy for pancreatic cancer in Japan.

## Graphical abstract

Utilization pancreatic cancer chemotherapy

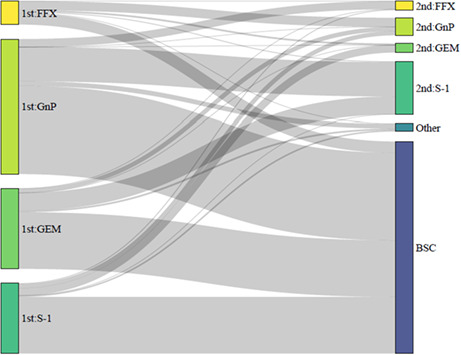

## 1 INTRODUCTION

Pancreatic cancer is one of the leading causes of cancer death in developed countries and one of the most fatal cancers in the world.^1^ In Japan, pancreatic cancer has the fourth highest site‐specific cancer mortality rate. There are more than 39,000 affected patients and more than 34,000 deaths per year, with the numbers increasing every year. Pancreatic cancer also has a worse prognosis than other cancers, with a 5‐year survival rate of 9.9% in Japan.^2^ The reported 5‐year relative survival rates by cancer stage are 42.9% for Stage I, 16.8% for Stage II, 7.4% for Stage III, and 1.5% for Stage IV. The specificity of pancreatic cancer symptoms is low, early detection is thus difficult. According to the Japanese cancer registry, only 1.1% and 24.5% of patients were diagnosed at stage 0 and stage I, respectively, with few initial symptoms.^3^

The treatment options for pancreatic cancer and their underlying rationales were set out in the 2019 Japanese clinical practice guidelines for pancreatic cancer according to cancer stage.^4^ In Stage 0–II cases, surgical treatment methods are mainly recommended. Surgical resection is the only potentially curative treatment, but it is estimated that only 20%–30% of all initial pancreatic tumors are resectable, and recurrence is extremely common even after resection. Adjuvant chemotherapy is recommended for resectable tumors. In recent years, several randomized control trials (RCTs) have shown improved survival with adjuvant chemotherapy compared to gemcitabine monotherapy (GEM), which was once the gold standard of chemotherapy.^5^, ^6^ For locally advanced pancreatic cancer classified as Stage III, treatment with a combination of radiotherapy and chemotherapy or either of them is mainly conducted to prolong life and improve quality of life. Although the evidence remains scarce, several regimens for the combination of chemotherapy and radiotherapy have been suggested to be beneficial in locally advanced unresectable pancreatic cancer based on the results of case‐series studies, mainly Phase I and II trials.^7^, ^8^ In cases of distant metastases classified as Stage IV, chemotherapy alone is the recommended treatment regimen. To confirm the benefit of chemotherapy, several trials have been conducted comparing chemotherapy with best supportive care (BSC) in patients with unresectable pancreatic cancer, with a significant survival benefit with chemotherapy reported in relevant meta‐analyses.^9^

The 2019 clinical practice guidelines for pancreatic cancer in Japan recommended or suggested five chemotherapy regimens for metastatic pancreatic cancer, namely FOLFIRINOX (FFX), gemcitabine plus nab‐paclitaxel (GnP), GEM, S‐1 (tegafur, gimelacil, and oteracil potassium combination), and gemcitabine plus erlotinib (GEM + Erlo).^4^ FFX or GnP is recommended as first‐line treatment for patients with low performance status (PS) based on the intensity of adverse events. The other three regimens are recommended for patients unsuitable for FFX or GnP due to high PS or advanced age. In comparison to GEM, overall survival with FOLFIRINOX was prolonged statistically significant in a global Phase III trial (hazard ratio [HR]: 0.57, 95% confidence interval [CI]: 0.45–0.73).^10^ However, the risk ratios for febrile neutropenia and neuropathy exceeded 1.5, and careful attention should be paid to adverse events. While in a Phase II single‐arm study conducted in Japan, FOLFIRINOX showed a survival of 10.7 months, similar to that reported in global clinical trials, febrile neutropenia occurred at a high rate of 22.2%.^11^ One treatment regimen that has mitigated FFX‐related adverse events by removing the rapid administration of fluorouracil and reducing the dose of irinotecan is modified FOLFIRINOX (mFFX). In a Japanese Phase II single‐arm study, mFFX showed an efficacy similar to that of FOLFIRNOX and a lower risk of adverse events.^12^ Similarly, GnP showed a statistically significant overall survival advantage over GEM in a global Phase III trial (HR: 0.72, 95% CI: 0.62–0.83).^13^ However, the risk ratios for interstitial pneumonia, sensory neuropathy, and fatigue exceeded 1.5, and appropriate action is also required regarding toxicity. In a Japanese Phase II single‐arm study of GnP, the median survival was 13.5 months, which was significantly higher than that reported in the abovementioned global Phase III trial.^14^ These results suggested that, despite notable concerns about adverse events, FFX (including mFFX) and GnP are treatment regimens that can significantly prolong overall survival and are thus recommended as first‐line chemotherapy for patients with high PS. S‐1, which, to date, has only been approved in Japan and South Korea, is widely used as first‐line chemotherapy for pancreatic cancer, due to its convenience of treatment by oral administration.^15^ S‐1 is used to the same extent as GEM, as direct comparisons have shown its non‐inferiority in overall survival (HR: 0.96) and a similar frequency and extent of adverse events.^16^ S‐1 is often recommended in patients at high risk of interstitial pneumonia, a typical side effect of GEM. Finally, GEM + Elro have demonstrated a significant overall survival advantage over GEM in a Phase III study (HR: 0.82, 95% CI: 0.69–0.99).^17^ However, the risk ratio of interstitial pneumonia for GEM + Elro to GEM is 1.72, so care should be taken in selecting pancreatic cancer patients for GEM + Elro as first‐line chemotherapy.

Thus, there are a variety of treatment options for systemic chemotherapy for pancreatic cancer, and further development can be expected in the future through national and international clinical trials. Nevertheless, the currently available treatment regimens are selected according to disease status and patient needs in the clinical setting, and it is unclear whether treatment guidelines are necessarily followed in clinical practice. To date, there are no large‐scale observational studies on the use of systemic chemotherapy in the treatment of pancreatic cancer that reflect clinical practice in Japan. In addition, while to some extent, efficacy and safety data are available from RCTs, there are no studies investigating the impact of a series of pancreatic cancer treatments on healthcare costs, including medicine costs. Hence, the economic impacts of the various treatment regimens and their differences are unclear.

Therefore, this study used medical management data on inpatients and outpatients treated at diagnosis procedure combination (DPC) hospitals in Japan to clarify the actual practice in treating pancreatic cancer patients with systemic chemotherapy in Japan and to estimate the associated direct medical costs.

## 2 METHODS

### 2.1 Data source

This study was a retrospective observational study and was conducted using the medical claims database possessed by Medical Data Vision Co, Ltd (MDV). The database contains administrative data and laboratory results for inpatients and outpatients. Specifically, the used data included anonymized patient ID, patient gender, year of birth, department visited, dates of medical services received, diagnosis codes, hospital admissions, medical procedures and laboratory orders, and surgery and prescription information. The data from April 2008 to December 2018 were analyzed. The follow‐up period was from the date of the first confirmed diagnosis of pancreatic cancer to the date of death or the end of the observation period.

### 2.2 Inclusion and exclusion criteria

The subjects in this study were pancreatic cancer patients who had never undergone surgery or radiotherapy following a confirmed diagnosis of pancreatic cancer. The inclusion criteria were as follows: (1) patients with at least one claim with definitive diagnosis of pancreatic cancer (cd10code = C25x) during the study period, (2) patients with any claim more than 28 days before the date of first confirmed diagnosis of pancreatic cancer (lookback period), (3) patients who received chemotherapy treatment recommended for pancreatic cancer within 60 days of the date of first confirmed diagnosis, and (4) patients with first confirmed diagnosis after January 1, 2015. Regarding the fourth reason for setting the inclusion criteria, the most recent GnP among the treatment regimens recommended or proposed as first‐line chemotherapy in Japanese pancreatic cancer practice guideline 2019 was approved in December 2014. Therefore, to ensure that the analysis reflects current treatment options, the analysis period of this study was defined as from January 1, 2015 or later. The exclusion criteria were as follows: patients who underwent surgery or radiotherapy after the date of first confirmed diagnosis of pancreatic cancer.

To determine the baseline demographic and clinical characteristics of the included patients, data on age, sex, weight, BMI, cancer stage, type of cancer, year of first confirmed diagnosis, clinical departments, complications (chronic pancreatitis, Type 2 diabetes mellitus), and history of multiple cancers were also collected. The baseline was defined as the date of the first confirmed diagnosis of pancreatic cancer in the injury code.

### 2.3 Regimens, time periods, and medical costs

The types of regimen used in first‐line chemotherapy were determined for anticancer drugs that were indicated as systemic chemotherapy for pancreatic cancer and whose prescription was initiated within 6 months of a confirmed diagnosis and administered within 28 days of the first prescription date. However, FFX includes at least two medications from the standard regimen, and GnP includes nab‐paclitaxel. All regimens that did not fit the recommended regimen were classified as “other.” The types of regimen used in second‐line chemotherapy were determined for all anticancer drugs indicated for pancreatic cancer administered within 28 days of the initial prescription date.

**FIGURE 1:**
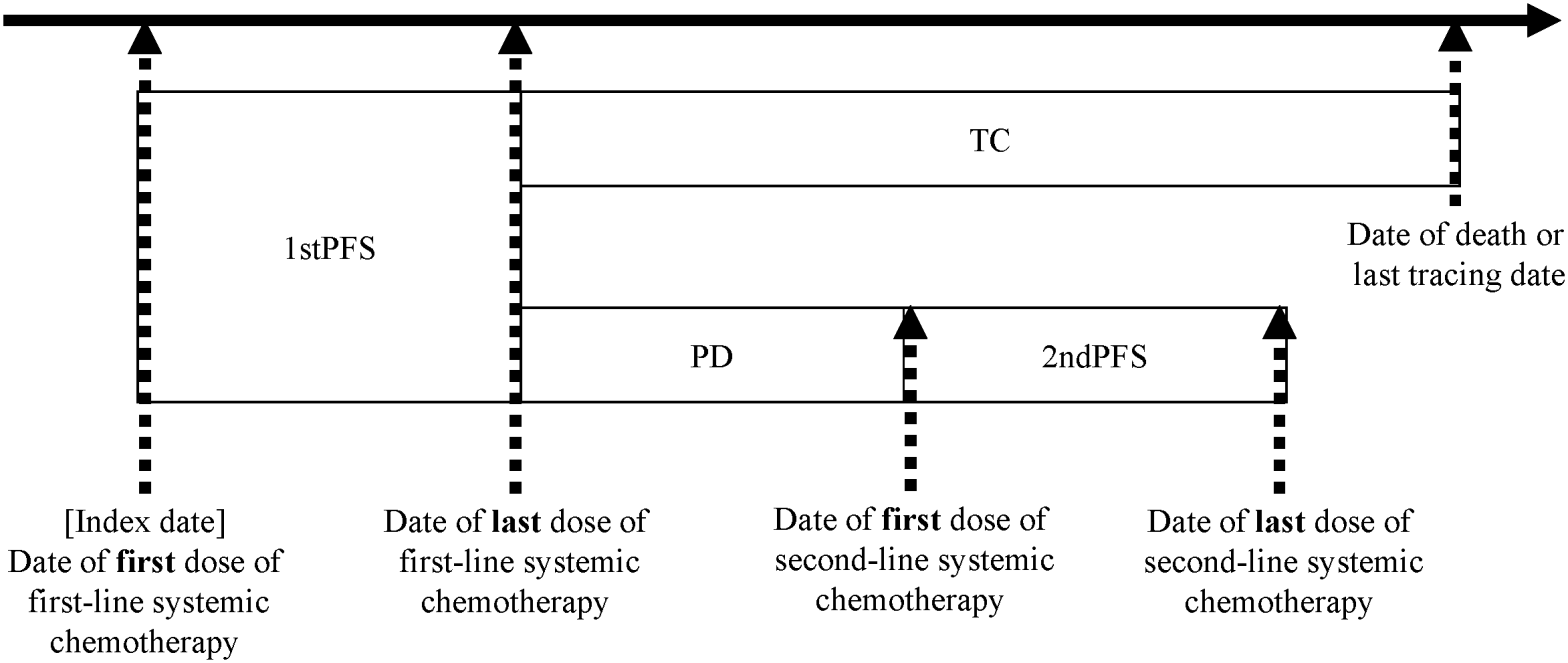
Treatment flowchart and definition of each episode. 1stPFS, first‐line progressive‐free survival; 2ndPFS, second‐line progressive‐free survival; PD, progressive disease; TC, terminal care.

The index date was defined as the date of the first prescription of systemic chemotherapy indicated for pancreatic cancer. The end date was the last prescription date of the systemic chemotherapy. If the indicated systemic chemotherapy had an oral administration, the treatment end date was defined as the last prescription date for the oral drug plus the number of days divided by the standard daily dose or the last tracking date in the record, whichever was earlier. The standard dose was determined by the handbook of cancer chemotherapy regimens in Japan, sixth edition.^18^ If some of the primary chemotherapy agents were discontinued and the entire or part of the treatment regimen was subsequently restarted, the subsequent treatment regimen was considered the same first‐line chemotherapy. The time period from the index date to the last prescription date of the first‐line chemotherapy was defined as first‐line progressive‐free survival (1stPFS) (Figure 1).

The period from the last day of 1stPFS to the start of the second‐line chemotherapy regimen or the last follow‐up date in the record, whichever was earlier, was defined as first‐line progressive disease (1stPD) and terminal care (TC), respectively. Switching was defined as prescribing a pancreatic cancer treatment regimen different from the primary chemotherapy treatment regimen. However, if all or part of the primary chemotherapy treatment regimen was discontinued and all or part of the drugs were subsequently restarted, the first‐line chemotherapy was considered ongoing. The period from the date of the first second‐line chemotherapy prescription to the date of the last prescription was defined as second‐line progressive‐free survival (2ndPFS). Overall survival (OS) was defined as the period from the date of the first prescription of first‐line chemotherapy to the date of death or last follow‐up, whichever was earlier.

Medical costs were estimated month by month during all episodes (1stPFS, TC, PD, and 2ndPFS) to capture changes over the treatment period. Using an episode lasting less than 1 month to estimate costs could lead to over‐ or under‐estimation of healthcare costs per month. Therefore, we only included in our healthcare cost estimates time periods of at least 1 month. The total costs per episode, including OS, were calculated for reference. Tracking periods and total costs per episode were also disaggregated for reference purposes. The exchange rate was set at 1 USD = 114.59 JPY (as of April 2019).

### 2.4 Endpoints

In this study, we analyzed the following endpoints to assess the use status of the systemic chemotherapy regimens recommended in Japan and to estimate the associated direct medical costs. The endpoints were analyzed for each of the treatment regimens recommended or proposed for systemic chemotherapy in Japanese practice guidelines, including FFX, GnP, GEM, and S‐1 (these four regimens are hereafter referred to as “recommended regimens”). Although GEM + Erlo is one of the drug combinations suggested in the guidelines, it was assumed that its limited number of prescriptions would make it difficult to interpret the results, hence, GEM + Erlo was included in the “other” treatment regimens group.

The cost estimates for each recommended regimen are shown in Table 1. The following four‐episode categories were used (Figure 1): (1) progression‐free survival (1stPFS) during first‐line chemotherapy, (2) progressive disease (PD) and (3) terminal care (TC) during first‐line chemotherapy, and (4) progression‐free survival during second‐line chemotherapy (2ndPFS). The maximum number of follow‐up months for the estimation of the monthly medical health care costs was set to 6 months, as patients in the 1stPD category were expected to enter the 2ndPFS category over a shorter time period.

**TABLE 1:** Patient characteristics.

Category	FFX		GnP		S‐1		GEM		Other	
	*N*	%	*N*	%	*N*	%	*N*	%	*N*	%
All patients	319	100	1838	100	962	100	1100	100	295	100
Age (year)										
Median (range)	65 (29–82)		71 (34–89)		75 (24–92)		76 (39–91)		71 (20–87)	
Age ≧ 65	161	50.5	1492	81.2	822	85.5	991	90.1	237	80.3
Age < 65	158	49.5	346	18.8	140	14.6	109	9.9	58	19.7
Sex										
Male	206	64.8	1132	61.6	572	59.5	628	54.4	187	63.4
Female	113	35.2	706	38.4	390	40.5	526	45.6	108	36.6
Weight (kg)										
*N*	318	99.7	1787	97.2	922	95.8	1078	98.0	291	98.6
Mean (SD) (kg)	58.5 (12.0)		56.3 (11.3)		54.2 (10.5)		53.4 (10.1)		56.0 (11.1)	
BMI (kg/m^2^)										
*N*	318	99.7	1787	97.2	920	95.6	1078	98.0	290	98.3
BMI < 18.5	50	15.7	283	15.8	149	16.2	199	18.5	54	18.3
18.5 ≤ BMI < 25	218	68.6	1227	68.7	654	71.1	734	68.1	196	66.4
BMI ≤25	50	15.7	277	15.5	117	12.7	145	13.5	40	13.6
Cancer stage										
*N*	308	96.6	1726	93.9	778	80.9	1014	92.2	241	81.7
0	1	0.3	5	0.3	5	0.6	3	0.3	0	0.0
I	3	1.0	30	1.7	42	5.4	18	1.8	7	2.4
II	8	2.6	89	5.2	107	13.8	68	6.7	11	3.7
III	31	10.1	165	9.6	121	15.6	103	10.2	19	6.4
IV	219	71.1	1195	69.2	346	44.5	661	65.2	169	57.3
Other/unknown	46	14.9	242	14.0	157	20.2	161	15.9	38	12.9
Location										
Head	131	41.1	756	41.1	412	42.8	460	41.8	96	32.5
Body	56	17.6	436	23.7	162	16.8	230	20.9	42	14.2
Tail	49	15.4	299	16.3	80	8.3	157	14.3	34	11.5
Head + Body	14	4.4	37	2.0	16	1.7	18	1.6	1	0.3
Body + Tail	18	5.6	142	7.7	37	3.9	76	6.9	16	5.4
Head + Tail	0	0.0	9	0.5	0	0.0	5	0.5	1	0.3
Head + Body + Tail	1	0.3	3	0.2	2	0.2	1	0.1	2	0.7
Unknown	35	11.0	139	7.6	203	21.1	130	11.8	56	19.0
Other	15	4.7	17	0.9	50	5.2	23	2.1	47	15.9
Year of first confirmed diagnosis (The denominator for the percentage was the total number of patients per year)										
2015	48	5.8	218	26.3	284	34.2	203	24.5	77	9.3
2016	64	6.3	353	34.5	281	27.4	254	24.8	72	7.0
2017	83	6.8	547	44.5	265	21.6	264	21.5	69	5.6
2018	124	8.7	720	50.3	270	18.9	241	16.8	77	5.4
Clinical departments										
Internal medicine	86	27.0	509	27.7	224	23.3	313	28.5	55	18.6
Gastroenterology	78	24.5	566	30.8	209	21.7	351	31.9	59	20.0
Surgery	65	20.4	268	14.6	303	31.5	176	16.0	97	32.9
Gastroenterology in general	44	13.8	343	18.7	117	12.2	165	15.0	48	16.3
Oncology	23	7.2	77	4.2	16	1.7	36	3.3	8	2.7
Digestive surgery	6	1.9	33	1.8	62	6.4	15	1.4	5	1.7
Hepatobiliary and pancreatic Surgery	11	3.5	18	1.0	13	0.0	5	0.5	1	0.3
Gastroenterology surgery	0	0.0	2	0.1	4	0.4	6	0.6	0	0.0
Other	6	1.9	22	1.2	14	1.5	33	3.0	22	7.5
Chronic pancreatitis										
No	286	89.7	1661	90.4	857	89.1	1014	92.2	257	87.1
Yes	33	10.3	177	9.6	105	10.9	86	7.8	38	12.9
Type II diabetes mellitus										
No	183	57.4	1015	55.2	574	59.7	638	58.0	180	61.0
Yes	136	42.6	823	44.8	388	40.3	462	42.0	115	39.0
History of multiple cancers										
*N*	316	99.1	1731	94.2	822	85.5	1025	93.2	274	92.9
No	258	81.7	1601	92.5	604	73.5	872	85.1	161	54.6
Yes	58	18.4	130	7.5	218	26.5	153	14.9	113	38.3

The shares of used healthcare resources were estimated for a maximum of 3 months based on the following six categories: (1) administration, (2) medicines, (3) procedures, (4) examinations, (5) hospitalization, and (6) other. Each category was identified and analyzed based on the following clinical identification codes: administration: 10–19, medicines: 20–29 and 30–39, procedures: 40–49 and 50–59, examinations: 60–69 and 70–79, hospitalization: 90–99, and other: all other practice identification codes.

The endpoints were as follows: (1) number and percentage of patients receiving first‐line chemotherapy for each recommended regimen, (2) number and proportion of patients receiving second‐line chemotherapy for each recommended regimen, (3) trends in monthly medical health care costs by duration for each recommended regimen, (4) percentage change in monthly medical health care costs in each health resource category by episode for each recommended regimen, and (5) total follow‐up time and total costs by episode for each recommended regimen.

### 2.5 Statistical analysis

Since the purpose of this study is to clarify the actual situation of treatment contents and medical expenses of systemic chemotherapy for pancreatic cancer in Japan, the analysis of this study is the descriptive statistics, and the clear sample size is not set in this research. We calculated summary statistics for each group of treatment regimens used in primary chemotherapy for the items age, sex, weight, BMI, cancer stage, type of cancer, year of first confirmed diagnosis, clinical departments, complications, and history of multiple cancers as patient characteristics. Also, the summary statistic was calculated similarly for the set treatment pattern and the endpoint of the medical expense. SAS version 9.4 (SAS Institute) and R version 3.6.3. were used for all analyses in this study.

## 3 RESULTS

### 3.1 Patient selection

**FIGURE 2:**
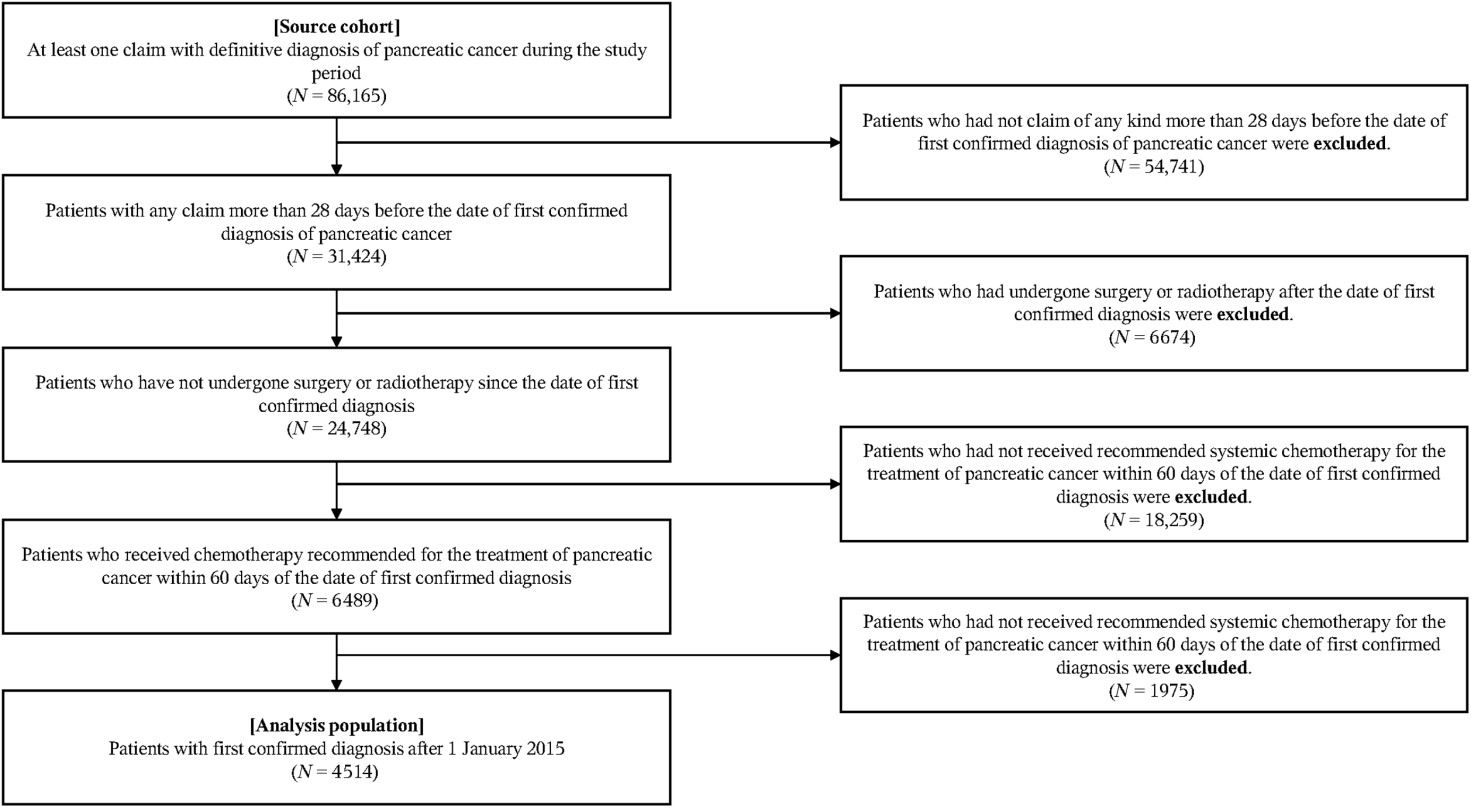
Patient flow diagram.

Of the 86,165 patients with a confirmed diagnosis of pancreatic cancer between April 2008 and December 2018, 31,424 patients received some medical treatment more than 28 days before their first confirmed diagnosis of pancreatic cancer. Of these, 24,748 patients had not undergone surgery or radiotherapy since the confirmed diagnosis. Of these, 6489 patients were using drugs for pancreatic cancer within 60 days of the date of confirmed diagnosis. Finally, we selected 4514 patients with a first confirmed diagnosis after January 1, 2015, as our analysis population (Figure 2).

### 3.2 Patient characteristics

Table 1 shows patient characteristics by first‐line chemotherapy treatment regimen. The largest number of patients received GnP as first‐line chemotherapy (1838 patients). This was followed by GEM with 1100 patients, S‐1 with 962 patients, and FFX with 319 patients. Age ranged from 20 to 92 years, with FFX being administered to the youngest population in terms of median age (65 years, range 29–82) and proportion of patients under 65 years (49.5%). For the other regimens, the median age and age range increased gradually, with 71 years (24–92) for GnP, 75 years (24–92) for S‐1, and 76 years (39–91) for GEM. Moreover, 81.2% and 78.8% of patients treated with FFX and GnP had Stage III or IV cancer, respectively, while cancer stage was other/unknown in 15%–20% of the cases. The head was the most common location for all treatment regimens (40%), followed by the body and tail. The proportions among all treatment regimens of S‐1 and GEM decreased from 34.2% to 18.9% and from 24.5% to 16.8% between 2015 and 2018, respectively, while the proportions of FFX and GnP increased from 5.8% to 8.7% and from 26.3% to 50.3%. There were similar trends between groups across clinical departments, complications, and patient history.

### 3.3 Use of chemotherapy treatment regimens

**FIGURE 3:**
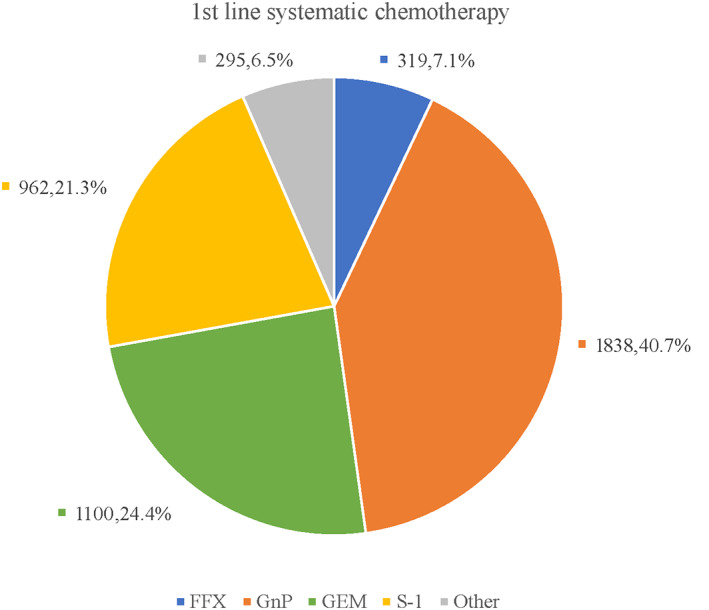
Proportion of treatment regimens prescribed in first‐line chemotherapy.

**FIGURE 4:**
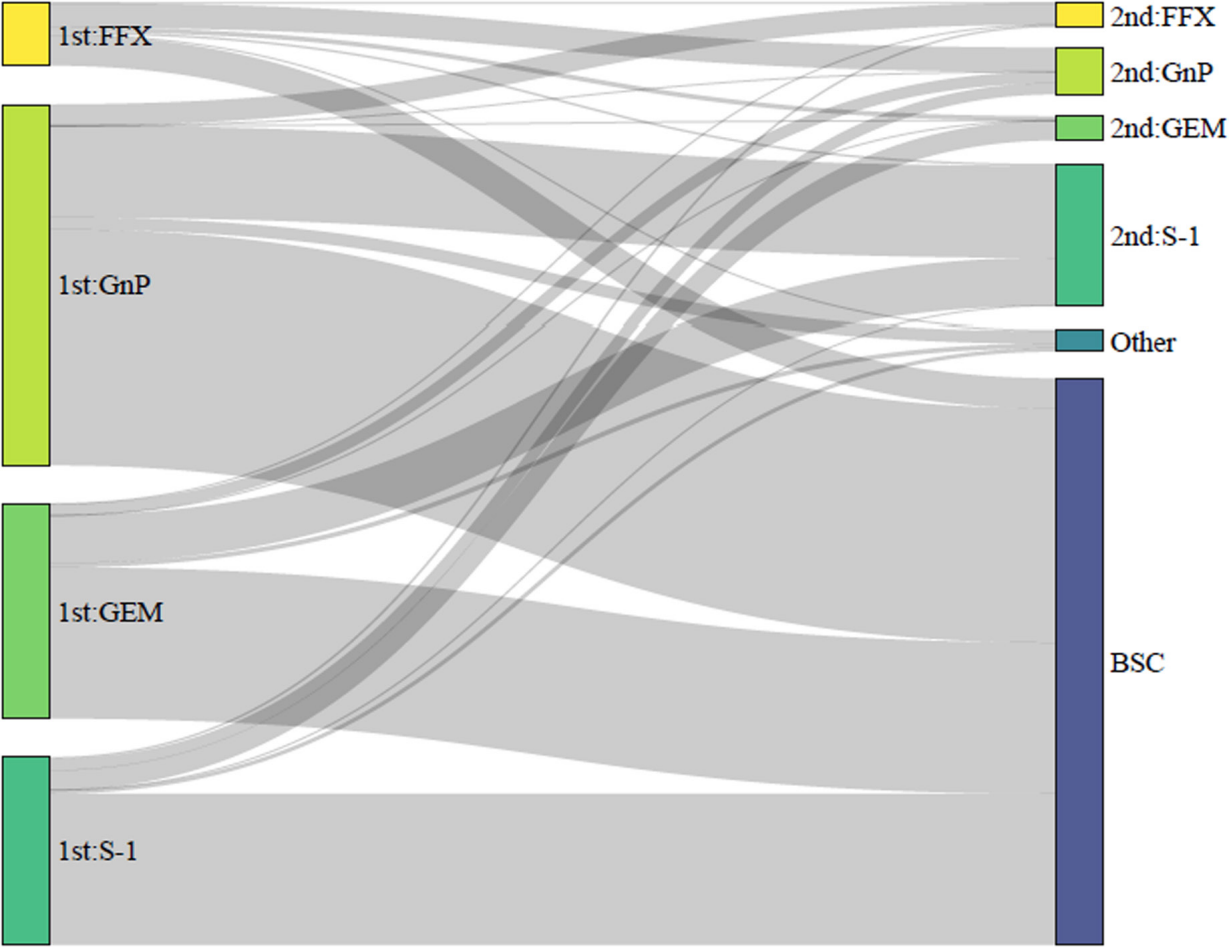
Flowchart of treatment options after the end of first‐line chemotherapy. BSC, best supportive care; FFX, FOLFIRINOX; GEM, gemcitabine monotherapy; GnP, gemcitabine plus nab‐paclitaxel.

Figure 3 shows the number and proportion of patients by first‐line chemotherapy treatment regimen. The most common treatment regimen was GnP (40.7%), followed by GEM (24.4%), S‐1 (21.3%), and FFX (7.1%). We also checked treatment status after completion of first‐line chemotherapy in the FFX, GnP, GEM, and S‐1 arms (Figure 4; Table S1). Of the patients who received first‐line chemotherapy, 31.3% were transferred to second‐line chemotherapy, and 68.7% were transferred to BSC. The highest proportion of patients opting for BSC was in the S‐1 arm at 80.5% (774 patients). This was followed by 70.3% (773 patients) in the GEM arm, 65.2% (1198 patients) in the GnP arm, and 48.3% (154 patients) in the FFX arm. The most common second‐line chemotherapy in the FFX arm was GnP (75.2%), with S‐1 (73.3%) in the GnP arm, S‐1 (74.7%) in the GEM arm, and GEM (53.8%) in the S‐1 arm. Less than 10% of treatment options in each arm were other than the recommended regimens for second‐line chemotherapy.

### 3.4 Medical health care costs

Table 2 presents summary statistics of medical costs per month for first‐line chemotherapy by episode and recommended regimen. For 1stPFS, medical costs at month 1 were the highest across all drug regimens, with median values of 6813 USD, 6125 USD, 3032 USD, and 1196 USD in GnP, FFX, GEM, and S‐1 treatments, respectively. Medical costs per month in 1stPFS gradually decreased from Month 2 onward, but the ranking of the treatment regimens remained the same, with GnP being the most expensive and S‐1 the least expensive. This trend was similar between the TC, 1stPD, and 2ndPFS periods. In addition, TC patients, a group that does not move to 2ndPFS after the end of 1stPFS, tended to have higher median medical costs than 1stPD patients, a group whose treatment progresses to 2ndPFS. However, when looking at the cumulative costs in OS from the start date to the last follow‐up date, the FFX arm had the most prolonged median follow‐up period of 215 days, which also resulted in the highest median total healthcare costs of 28,752 USD (Table S2).

**TABLE 2:** Estimated monthly health care cost for analysis population during follow‐up period (USD).

Period	Month	FFX						GnP					
		*N*	Mean	Std	Min	Median	Max	*N*	Mean	Std	Min	Median	Max
1stPFS	1	257	6634	2730	1347	6125	14,818	1452	7060	2647	1151	6813	18,634
	2	192	3498	1963	9	3013	10,617	1176	3994	2157	6	3590	19,015
	3	150	3618	2374	86	3024	13,341	964	4028	2327	68	3798	31,645
	4	126	3138	2457	50	2615	17,052	789	3503	1990	89	3116	15,238
	5	96	2954	2065	109	2284	12,190	662	3299	1849	17	2926	13,944
	6	75	3175	2085	230	2411	9045	527	3500	2139	106	3244	23,798
TC	1	69	6680	4581	635	5541	19,656	447	6708	4702	428	6438	37,718
	2	39	4904	4643	64	4575	16,546	237	4857	4423	6	4199	32,017
	3	26	3393	4845	6	740	15,629	132	4228	6058	30	2927	58,460
	4	20	3392	3875	60	1580	11,041	74	3869	4785	29	881	16,616
	5	17	2060	3141	34	595	11,564	59	2915	3984	14	593	19,390
	6	10	3034	3762	7	892	11,320	37	2705	3314	22	931	14,189
1stPD	1	47	5557	4562	858	4084	21,939	131	5184	3489	278	5081	17,470
	2	16	3007	3899	89	1309	12,520	34	2990	3435	9	1961	15,971
	3	4	5065	6528	266	2798	14,397	13	1666	2790	90	543	10,304
2ndPFS	1	103	5901	3230	1014	5033	20,502	202	5885	2304	1835	5427	13,338
	2	79	3083	1938	490	2685	8965	175	3645	2003	274	3264	11,504
	3	57	3591	2794	695	2743	16,365	145	3907	1820	143	3732	10,626
	4	41	2820	2206	551	1953	11,516	117	3300	2110	247	2906	12,484
	5	33	2586	1722	31	2080	7222	110	3201	2299	251	2831	15,823
	6	21	2667	1851	347	1933	7831	92	3831	3151	307	3175	19,486

Period	Month	GEM						S‐1					
		*N*	Mean	Std	Min	Median	Max	*N*	Mean	Std	Min	Median	Max
1stPFS	1	773	3732	2798	470	3032	24,145	721	2218	2249	147	1196	16,585
	2	564	1965	2262	28	1096	15,846	584	1428	2091	15	764	20,666
	3	417	1861	1902	42	1184	14,065	454	1353	1751	15	894	20,757
	4	333	1799	2314	134	1072	26,344	351	1316	2331	6	627	21,099
	5	263	1546	1724	50	994	11,525	307	1170	1356	31	804	12,925
	6	204	1577	1522	279	1073	8584	256	1412	2378	39	822	22,243
TC	1	381	5294	4247	215	4926	30,559	446	2825	3310	12	1215	30,670
	2	196	4661	4638	37	4019	25,419	300	3034	3903	9	963	26,456
	3	105	3971	4401	6	2444	23,940	221	2752	4509	6	554	23,207
	4	65	3243	3660	6	1711	15,598	173	2330	3316	6	618	15,930
	5	42	3229	3988	10	879	16,870	135	2077	3172	6	506	14,602
	6	32	2885	4145	6	571	18,469	125	2091	3662	11	460	21,286
1stPD	1	42	4561	3360	309	4406	14,272	118	1942	2448	334	999	13,988
	2	10	5985	8185	6	3360	26,979	44	1582	2558	106	448	10,307
	3	6	6297	8777	306	1420	21,086	24	1761	3245	84	379	11,021
2ndPFS	1	82	3089	2132	420	2538	9590	478	2117	2072	310	1292	13,440
	2	66	1575	1451	78	1049	6782	341	1486	1899	36	859	13,310
	3	44	2174	2726	478	1182	15,451	244	1514	1700	45	1022	10,591
	4	34	1649	1901	115	1020	8725	190	1168	1629	24	656	10,260
	5	25	1634	1818	652	1075	9314	140	1362	1908	158	929	16,603
	6	19	1762	2234	530	1164	10,611	105	1156	1369	148	878	10,423

**FIGURE 5:**
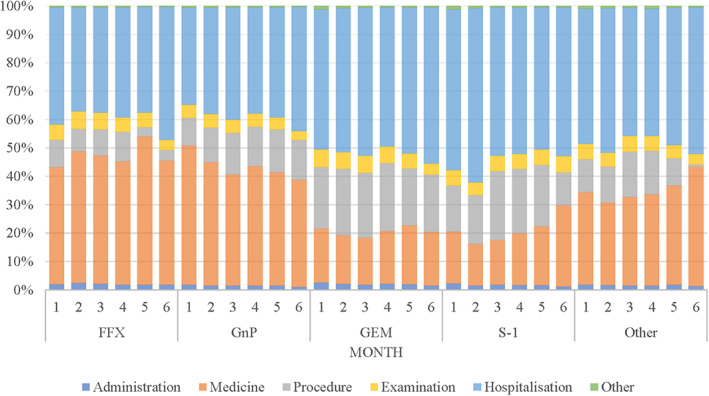
Distribution of monthly mean costs during progression‐free survival in first‐line chemotherapy by health resource category. FFX, FOLFIRINOX; GEM, gemcitabine monotherapy; GnP, gemcitabine plus nab‐paclitaxel.

Figure 5 shows the percentage change in monthly medical expenditure by treatment regimen and medical resource category during 1stPFS. In the FFX and GnP arms, the largest proportion of monthly medical costs was spent in hospitalization (FFX [Max–Min]: 41%–37%, GnP: 40%–34%) or medicine costs (FFX: 51%–42%, GnP: 49%–38%). Hospitalization costs was also most common in the GEM and S‐1 arms (GEM: 55–49, GnP: 61%–50%). The stacked sum of the average monthly medical costs by treatment regimen in 1stPFS by medical resource category showed that medical costs for FFX and GnP remained higher than those in the GEM and S‐1 groups, even as absolute values, indicating that drug costs had a larger impact on monthly medical costs (Figure S1).

The highest percentage of patients who started a first‐line chemotherapy treatment regimen completed it within 1 month (29.7%), with many patients going through each treatment regimen for at least 1 month. The proportion of patients who completed 1stPD less than 1 month after completing first‐line chemotherapy was the highest in the GEM group (87.2%) and the lowest in the FFX group (71.5%) (Table S3). It was also observed that many patients started second‐line chemotherapy in less than 1 month.

## 4 DISCUSSION

Based on MDV cohort data from 86,165 patients with a confirmed diagnosis of pancreatic cancer from April 2008 to December 2018, this study investigated the actual use of the treatment regimens recommended in Japan as systemic chemotherapy up to the second‐line treatment and estimated the related medical costs. The total number of pancreatic cancer patients in Japan from 2008 to 2018 was 393,534, with 22% of the acute‐care hospitals in Japan being covered by MDV in the data period. When multiplying the total number of patients affected by pancreatic cancer in Japan by the MDV coverage rate, the result of 865,771 was found to closely reflect the cohort data used in this study,^19^, ^20^, ^21^, ^22^ ensuring its representativeness of pancreatic cancer patients in acute‐care hospitals in Japan.

A wide range of age groups were selected in this study, from young to elderly patients. The age and gender of patients in each treatment regimen group were generally consistent with the characteristics of patients affected by pancreatic cancer in Japan. In addition, the median age and age range suggest that FFX and GnP regimens are used in younger populations than S‐1 and GEM. The tendency to use FFX and GnP in relatively young and physically fit patients could be due to their higher efficacy but lower tolerability compared to S‐1 and GEM. On the other hand, there were similar trends between groups regarding clinical departments, comorbidities, and medical history, which were other potential factors influencing treatment choice.

Although GnP was most commonly used as first‐line chemotherapy, FFX had the lowest use rate among the Japanese recommended regimens for systemic chemotherapy. This may be attributed to the very high toxicity of FFX. In a single‐arm Phase II trial of FFX in Japanese patients, the median survival was 10.7 months, comparable to that in overseas Phase III trials. However, febrile neutropenia, a known severe adverse event, occurred in 22.2% of patients.^11^ In addition, a global Phase III clinical trial reported a risk ratio of more than 1.5 for febrile neutropenia and peripheral neuropathy for FFX compared to GEM.^12^ By contrast, although GnP has raised concerns about the seriousness of interstitial pneumonia in international Phase III trials, the rate of febrile neutropenia was 5.9% in a single‐arm Phase II trial in Japan.^13^, ^23^ This rate was lower than that for FFX. These results suggest that FFX tends to be used mainly in patients in good health due to the potential adverse events. Conversely, GnP may offer a better balance between efficacy and safety than FFX and is thus used in a broader range of patients as a first‐line treatment regimen for systemic chemotherapy in pancreatic cancer.

The most common conversion rates to BSC after first‐line chemotherapy were, in descending order, from S‐1, GEM, GnP, and FFX. In Japanese practice guidelines, S‐1 and GEM are first‐line treatment regimens for systemic chemotherapy in pancreatic cancer patients, but they are only proposed when FFX and GnP are not suitable due to disease status and age.^4^ This suggested that many patients who received S‐1 and GEM as first‐line chemotherapy would transition to BSC due to concerns about their physical condition. The chosen treatment in patients who progressed to second‐line chemotherapy tended to differ pharmacologically from the treatment regimen used in the first‐line chemotherapy. Patients who received FFX or GnP as first‐line chemotherapy were also more likely to choose the more potent treatment regimens FFX and GnP as second‐line chemotherapy than those who chose S‐1 or GEM as first‐line chemotherapy. In particular, 75.2% of patients in the FFX arm who progressed to second‐line chemotherapy received GnP. This proportion was more than double that in the S‐1 arm, which shares pharmacological mechanisms with FFX. This may be because patients in the FFX arm were younger, had relatively better disease status, and could be treated more aggressively than those in the S‐1 arm. The treatment choice for second‐line chemotherapy in the GnP and GEM arms suggested a similar trend.

Medical costs for each group were estimated per month for up to 6 months. For first‐line chemotherapy, the GnP arm showed the highest medical costs, while the S‐1 arm has the lowest costs. The higher costs in the GnP arm was thought to be due to the inclusion of nab‐paclitaxel, the most expensive recommended regimen for pancreatic cancer in Japan. In fact, medicine costs in the GnP arm represented around 40% of the total costs, which was a higher proportion than that in the GEM and S‐1 arms. Medicine costs in the GnP group were also the highest among the different treatment regimens in terms of total value. Some other factors possibly affecting medicine costs could be the need for additional drugs to deal with adverse events, but the total cost of medicines in the GnP arm was greater than that in the FFX arm, which is assumed to require more treatment for adverse events than GnP. These results suggest that the impact of anticancer drugs on medical costs in GnP‐treated patients is substantial. In the S‐1 arm, which corresponds to the least expensive treatment category, there appeared to be an increase in the shares of medical procedures, examinations, and hospitalization. However, the absolute values for these medical resource categories in the S‐1 arm were comparable to those of other drug regimen arms, as the total medical costs were lower than those in other recommended treatment regimens. In addition, regardless of the treatment regimen, medical costs in the first month of 1stPFS were higher than those in the second and later months of 1stPFS. This observation is likely due to increased inpatient treatment in the early stages of FFX and GnP treatment due to safety concerns, as well as the highest proportion of patients experiencing difficulties in continuing treatment after the first one or two cycles of treatment.

Medical costs in TC and 1stPD were the highest in the first month, with 40%–50% of patients reaching the last follow‐up date or the start of second‐line chemotherapy between the first and second months. This suggests that immediately after the last prescription date of first‐line chemotherapy, the consumption of healthcare resources temporarily increases due to worsening physical symptoms associated with adverse events. Medical costs for second‐line chemotherapy were the highest in the first month for similar reasons and remained stable thereafter. The transition to multiple treatment regimens for second‐line chemotherapy made it difficult to capture the differences in characteristics between groups. However, a greater proportion of patients in the FFX and GnP arms also opted for a more potent treatment regimen in second‐line chemotherapy than in the GEM and S‐1 arms, which may have resulted in higher medical costs than in the GEM and S‐1 arms.

There are several possible limitations to this study. The first is that the data used in this study are receipt data derived from DPC hospitals and therefore do not include data from non‐DPC hospitals or clinics. In addition, we could not track visits at more than one hospital, which means that some patients may have received treatment at other hospitals before what we defined as first‐line chemotherapy. On the other hand, we removed as many patients as possible who were transferred from other hospitals by including a lookback period before the initial diagnosis of pancreatic cancer. This method is frequently used in cancer surveys using commercially available claims data.^24^

Second, due to the properties of this dataset, information on the cancer stage can only be obtained for some patients. Also, the data are not necessarily continuous and its reliability is uncertain. Therefore, in this study, stratification analysis using the stage of cancer was not performed. Unlike in other countries, Japan has very limited claims databases covering all healthcare facilities. To further improve the generalizability and validity of our findings, we plan to use a more extensive database, such as the national database, in a similar future study.

Third, the study did not perform cost estimation by treatment regimen for second‐line chemotherapy because of concerns about the very small sample size. There were differences between the groups in first‐line chemotherapy regarding patient characteristics and the proportions of treatment options in second‐line chemotherapy. This may lead to a different impact of second‐line chemotherapy on treatment and medical costs depending on the treatment regimen of the primary chemotherapy, even if the treatment choice for second‐line chemotherapy remained the same. Studies using data from a larger cohort are warranted to estimate medical costs for each treatment regimen in second‐line chemotherapy.

Finally, this study estimated medical costs per month in pancreatic cancer patients who were able to continue systemic chemotherapy for at least 1 month. Therefore, medical costs were not estimated for patients whose systemic chemotherapy was terminated within 1 month due to toxic effects or sudden changes in disease status. On the other hand, the number of treatment cycles that can be performed in a month is only one cycle (two cycles for FFX), considering the standard administration schedule of the recommended regimens covered in this study. Patients who completed treatment during this period did not receive stable treatment. Therefore, it is considered necessary to estimate medical costs in a different population than patients who continue treatment for more than 1 month. These considerations suggest that future studies are warranted to further investigate the differences in total medical costs and medical categories between patients who terminate treatment early and those who can continue treatment for a more extended period, based on patient characteristics.

In this study, a survey of the actual use of systemic chemotherapy in pancreatic cancer, mainly for first‐line chemotherapy, and an estimation of the related medical costs was conducted using large‐scale Japanese receipt data. The results suggest that the systemic chemotherapy treatments used for pancreatic cancer in Japan reflect the recommendations and suggestions laid out in Japanese guidelines. Moreover, when comparing the recommendations to first use FFX and GnP, more patients used GnP, suggesting that the choice of treatment was based not only on its efficacy but also on its balance with safety. Furthermore, the medical costs associated with GnP treatment were higher than those of all the other treatment regimens recommended in Japan. In the future, further information that can be used for decision‐making on treatment regimens should be provided by cost‐effectiveness analyses of the treatment regimens recommended in Japan, considering not only their efficacy and safety but also their economic impact. Since the actual conditions of use and estimated medical costs obtained in this study reflect the clinical conditions in Japan, it is considered that this data can be used for such analysis.

## 5 CONCLUSION

The actual treatment patterns and direct medical costs of systemic chemotherapy for pancreatic cancer in Japan were clarified in this study. The results suggest that, although most treatment options generally complied with Japanese practice guidelines, treatment choice was not necessarily only based on efficacy, but also on the patient's condition and the impact of adverse events. On the other hand, as medical costs differed between treatments, a multifaceted evaluation also considering efficacy and safety is considered necessary in the future.

## AUTHOR CONTRIBUTIONS

**Yuki Takumoto:** Conceptualization (equal); data curation (equal); formal analysis (equal); investigation (equal); methodology (equal); project administration (equal); supervision (equal); validation (equal); visualization (equal); writing – original draft (equal). **Ryotaro Shibahara:** Data curation (supporting); formal analysis (lead); software (equal); validation (equal); visualization (equal); writing – review and editing (equal). **Hajime Asami:** Data curation (lead); formal analysis (lead); software (equal); validation (equal); visualization (equal); writing – review and editing (equal). **Manabu Akazawa:** Conceptualization (equal); funding acquisition (equal); methodology (equal); project administration (equal); resources (equal); software (equal); validation (equal); writing – review and editing (equal).

## CONFLICT OF INTEREST STATEMENT

Manabu Akazawa received honoraria and manuscript fees from Jansen, GSK, Takeda, and Shionogi. Other authors declare having no conflicts of interest directly relevant to the content of this article. Affiliations of Yuki Takumoto, Ryotaro Shibahara, and Hajime Asami are as of March 2023.

## ETHICS STATEMENT

This study was approved by the Research Ethics Committee of Meiji pharmaceutical university (permission number: 201902). Our study uses an anonymized claim database and does not apply to the availability of informed consent in clinical trials involving human participants.

## Supporting information


**Data S1.** Supporting Information.

## Data Availability

Not available because the datasets are commercial data of Medical Data Vision, Co., Ltd.
