# Supplementary material for: Treatment patterns and cost estimations of systemic chemotherapy for pancreatic cancer in Japan: A retrospective database study
Source: Cancer Med. 2023 May 18;12(13):14742–55. doi: 10.1002/cam4.6100 (PMC10358248; doi:10.1002/cam4.6100)
Supplement: Supplementary file 1 — Data S1. Supporting Information. [file CAM4-12-14742-s001.docx]

Supplementary Figure S1. Estimated monthly health care costs by health resource category in first-line chemotherapy

Supplementary Table S1. Distribution of treatment options after the end of first-line chemotherapy

| Treatment options after the end of first-line chemotherapy | ALL | | FFX | | GnP | | GEM | | S-1 | |
| --- | --- | --- | --- | --- | --- | --- | --- | --- | --- | --- |
|  | N | % | N | % | N | % | N | % | N | % |
| BSC | 2,900 | 68.7% | 154 | 48.3% | 1,198 | 65.2% | 773 | 70.3% | 774 | 80.5% |
| 2nd line chemotherapy - ALL | 1,319 | 31.3% | 165 | 51.7% | 640 | 34.8% | 327 | 29.7% | 188 | 19.5% |
| 2nd line chemotherapy – Individual regimen (The denominator for each regimen was “2nd line chemotherapy - ALL”) | | | | | | | | | | |
| FFX | 124 | 9.4% | - | - | 109 | 17.0% | 3 | 0.9% | 12 | 6.4% |
| GnP | 239 | 18.1% | 124 | 75.2% | - | - | 58 | 17.8% | 57 | 30.4% |
| GEM | 123 | 9.3% | 22 | 13.3% | 0 | 0.0% | - | - | 101 | 53.8% |
| S-1 | 725 | 54.9% | 12 | 7.3% | 469 | 73.3% | 244 | 74.7% | - | - |
| Other | 109 | 8.3% | 7 | 4.2% | 62 | 9.7% | 22 | 6.7% | 18 | 9.6% |

Supplementary Table S2. Calculated total health care costs and study periods

| Category | Outcome | FFX | | | | GnP | | | | GEM | | | | S-1 | | | |
| --- | --- | --- | --- | --- | --- | --- | --- | --- | --- | --- | --- | --- | --- | --- | --- | --- | --- |
|  |  | N | Median | Q1 | Q3 | N | Median | Q1 | Q3 | N | Median | Q1 | Q3 | N | Median | Q1 | Q3 |
| OS | Total Cost (USD) | 319 | 28,752 | 17,920 | 48,773 | 1,838 | 26,516 | 14,136 | 42,662 | 1,100 | 15,986 | 8,291 | 26,856 | 962 | 13,113 | 5,924 | 24,298 |
|  | Number of Days | 319 | 215 | 103 | 379 | 1,838 | 187 | 85 | 327 | 1,100 | 146 | 65 | 296 | 962 | 203 | 93 | 412 |
| 1stPFS | Total Cost (USD) | 319 | 13,604 | 7,319 | 23,336 | 1,838 | 15,722 | 8,198 | 27,794 | 1,100 | 5,568 | 2,854 | 10,624 | 962 | 3,771 | 1,720 | 8,602 |
|  | Number of Days | 319 | 91 | 41 | 175 | 1,838 | 104 | 40 | 204 | 1,100 | 64 | 28 | 151 | 962 | 84 | 21 | 176 |
| TC | Total Cost (USD) | 154 | 1,144 | 944 | 1,464 | 1,198 | 1,234 | 892 | 1,372 | 773 | 334 | 272 | 442 | 774 | 487 | 350 | 733 |
|  | Number of Days | 154 | 21 | 14 | 32 | 1,198 | 14 | 7 | 27 | 773 | 13 | 7 | 21 | 774 | 38 | 26 | 61 |
| 1stPD | Total Cost (USD) | 165 | 1,223 | 841 | 1,634 | 640 | 1,235 | 1,049 | 1,384 | 327 | 364 | 279 | 469 | 188 | 441 | 320 | 634 |
|  | Number of Days | 165 | 28 | 2 | 75 | 640 | 21 | 1 | 58 | 327 | 36 | 15 | 70 | 188 | 52 | 14 | 148 |

Supplementary Table S3. Distribution of patients across clinical conditions with a follow-up time of less than one month

| **Follow up period** | **FFX** | | **GnP** | | **GEM** | | **S-1** | |
| --- | --- | --- | --- | --- | --- | --- | --- | --- |
|  | N | % | N | % | N | % | N | % |
| 1stPFS | | | | | | | | |
| < 1 month | 62 | 19.4% | 386 | 21.0% | 327 | 29.7% | 241 | 25.1% |
| ≧ 1month | 257 | 80.6% | 1452 | 79.0% | 773 | 70.3% | 721 | 74.9% |
| TC | | | | | | | | |
| < 1 month | 85 | 55.2% | 751 | 62.7% | 392 | 50.7% | 328 | 42.4% |
| ≧ 1month | 69 | 44.8% | 447 | 37.3% | 381 | 49.3% | 446 | 57.6% |
| 1stPD | | | | | | | | |
| < 1 month | 118 | 71.5% | 509 | 79.5% | 285 | 87.2% | 347 | 83.2% |
| ≧ 1month | 47 | 28.5% | 131 | 20.5% | 42 | 12.8% | 70 | 16.8% |
